# 

**Published:** 1828-04

**Authors:** 


### CONTENTS OF THE SECOND VOLUME.

### NO. I.

### ESSAYS AND CASES.

A Discourse on the Causes, Effects, and Preventives of Intemperance, from Ardent Spirits. By DANIEL DRAKE, M. D. 9

REVIEWS AND BIOGRAPHICAL NOTICES.

Formulary for the preparation and employment of the Salts of Morphine, of Strichnine, Brucine, Cinchonine, Quinine, Emetine, Iodine and other new medicines. By F. MAJENDIE. - - - - - - - - - - 35

Observations on the efficacy of the White Mustard Seed in affections of the Liver, Internal Organs, and Nervous System. By CHARLES TURNER COOKE, Member of the Royal College of Surgeons. - - - - - - 50

MISCELLANEOUS INTELLIGENCE.

ANALECTIC.

On Amputation and the omission of Ligatures to the vessels. By Dr. L. KOCH. - - - - - - 55

Chorea cured with Venesection and Leeching. By M. LISFRANC. - - - - - - - - - - 57

On the distinction between Rheumatic Inflammation of the Heart and Pericarditis. By ROBERT ADAMS, Esq. - 58

ANALYTICAL.

Excision of the Tonsils. By A. H. HOSACK, M. D. - 59

Ammonia in the bites of Venomous Snakes. By Dr. MOORE. 60

Eruptions caused by Balsam Copavia. By T. HEWSON, M. D. 60

ORIGINAL.

Quarterly Report of the Weather and Diseases of Cincinnati. By the EDITOR. - - - - - - - - 60

Necrology. - - - - - - - - 64

NO. II.

ESSAYS AND CASES.

A Discourse on the Causes, Effects, and Preventives of Intemperance, in the use of Ardent Spirits. By DANIEL DRAKE, M. D. Concluded. - - - - - 65

Appendix on Alcohol. By the same. - - - - 87

### REVIEWS AND BIBLIOGRAPHICAL NOTICES.

Formulary for the preparation and employment of the new medcines Veratria, Hydrocyanic Acid, Delphinia, Iodine, &c. By F. MAJENDIE. Concluded. - - - - 92
Appendix of Tables of French and English Weights and Measures. By Dr. ATLEE. - - - - - 108
Observations and Experiments on Cupping-Glasses and Pressure in Poisoned wounds. By CASPAR WISTAR PENNOCK, M. D. - - - - - 109

### MISCELLANEOUS INTELLIGENCE.

### I. ANALECTIC.

Permanent Evidence of successful Vaccination. By. Dr. GREGORY. - - - - - - 113
Ununited Fracture Cured by Pressure. By Mr. BRODIE. 114

### II. ANALYTICAL.

Poisoning with Sulphuric Acid—Death of Mr. Ledyard. By the EDITOR. - - - - - 114

### III. ORIGINAL.

Winter Quarterly Report on the Diseases of Cincinnati, concluded—Treatment of Bronchitis. By the EDITOR. 116
To Subscribers, Correspondents and Editors. - - 120

### *NO. III.*

### ESSAYS AND CASES.

Cases Illustrative of the Effects of Iodine. By JEREMIAH H. BROWER, M. D. - - - - - - 121
An account of a Rupture of the Pylorus, with the appearances after death. By the EDITOR. - - - 126
Essay on Spontaneous Human Combustion. By M. MARC. Translated from the French. By the EDITOR. - 130
Notices of the Principal Mineral Springs of Kentucky and Ohio. By the EDITOR. - - - - - 142
Harrodsburgh Springs, - - - - 145
Rochester Spring, - - - - - 151
Olympian Springs, - - - - - 151
Blue Licks, - - - - - 156
Big Bone Lick, - - - - - 158
Yellow Spring, - - - - - 164

### MISCELLANEOUS INTELLIGENCE.

### I. ANALECTIC.

Peculiar Disease of the Heart, - - 167
Intestinal Invaginations, - - - - 171
Endermic Medications. - - - 173

### II. ORIGINAL.

Report on the Weather and Diseases of Cincinnati, in the Spring of 1828. By the EDITOR. - - - 174

Pulmonary Inflammations, Measles, Mumps. - 176

### NO. IV.

### ESSAYS AND CASES.

History of a Case of Ischuria successfully treated with the Cold Affusion. By Dr. Lewis Campbell. - - 177
A Case of Poisoning by Opium treated successfully with Cold Water. By Dr. John F. Brown. - - 179
An account of the beneficial Effects of Ergot in a case of Hydatids of the Uterus. By Dr. P. B. Jones. - - 181

### REVIEWS.

An Essay upon the Medical Properties of Plants compared with their Exterior Forms and their Natural Classification, By A. P. De Candolle. - - 183

### MISCELLANEOUS INTELLIGENCE.

I. ANALECTIC.

Communication between the Bloodvessels of the Uterus and the Placenta. - - - - - 209
New Views of the Fœtal Circulation, by Dr. H. F. Kilian. 209
Puerperal Peritonitis cured by Ice. - - - 210
Congenital absence of Sensation and Voluntary Motion in a female. - - - - - 211
State of the Spinal Marrow and Nerves in Tetanus, by M. Lepelletier. - - - - - 211
Mercury absorbed into the System. - - 212
Amputation of the Lower Jaw, by M. Lisfranc. - 213
Writ for a Medical Convention in 1830, by S. L. Mitchill, M. D. - . - - 213
Treatment of Poisoned Wounds, by M. Verniere, - 214
Death by the external application of Opium. - 215
Sounding the internal Ear in Deafness. - - 215

II. ORIGINAL.

Report on the Diseases of Cincinnati in the Spring of 1828, by the Editor, concluded. - - - - 216
Fractures of the Thigh, by the Editor. - - 219
Board of Health in Cincinnati. - - - 221
Splendid Engravings of American Birds, J. J. Audubon. 221
Lectures in the Medical College of Ohio. - 223
Lectures in the Medical Department of Transylvania University. - - - - - 224
To Subscribers. - - 224

### NO. V.

### ESSAYS AND CASES.

Editorial Address to the Physicians of the West and South.
Observations on Mr. Guthrie's Double Cataract Knife. By the Editor. - - - - - - 231
Practical observations on the Uterine Hœmorrhage, connected with Abortion and Parturition. By the Editor. 241

REVIEWS.

Observations on Expansibility as a Vital Property, and on the Influence of the Capillary Tissue, over the Circulation of the Blood, by HUGH L. HODGE, M. D. - - 257

MISCELLANEOUS INTELLIGENCE.

ANALYTICAL AND ORIGINAL.

1. Ununited Fracture cured by Pressure. By T. H. WRIGHT, M. D. - - - - 274
2. Salivation from Inhaling the Breath of a Salivated Patient. 276
3. Poisonous Expectoration in Phthisis Pulmonalis. - 277
4. Death of Professor Post. - - - 277
5. Resignation of Professor Godman. - - 278
6. Tribute of Respect to Dr. Holyoke. - - 278

*NO. VI.*

ESSAYS AND CASES.

Observations on the reciprocal propagation of Disease between the Thoracic and Abdominal Viscera. By the EDITOR. 281
Some Observations on Ulceration of the Kidneys; with cases. By JOHN C. WARREN, M. D. - - 290

REVIEWS.

1. An exposition of the Natural System of the Nerves of the Human Body. By CHARLES BELL, Professor of Anatomy, &c. &c. London, 1824.
2. Appendix to the Papers on the Nerves, republished from the Royal Society's Transactions. By CHARLES BELL, London, 1827. - - - - 307

MISCELLANEOUS INTELLIGENCE.

Notice to Subscribers. - - - 328
First District Medical Society of Ohio. - 328

*NO. VII.*

ESSAYS AND CASES.

Report of a case of Gunshot wound, in which five shot were lodged in the cavities of the Heart. By Dr. LEONARD RANDAL. - - - - - - 330
Wounds of the heart. Translated from the French, by the EDITOR. - - - - - - 333
Some account of a case of Softening of the Heart and Emphysema of the Lungs, with the morbid appearances after death, By the EDITOR. - - - - - - 337
Digested extracts from Lænnec, on the Chest:— -
1. On Emphysema of the Lungs. - - - 341
2. Pericarditis. - - - - - 356
3. Softening of the Heart. - - - 461

Notices concerning the Dengue:— - - -
1. Editorial remarks. - - - 363
2. Memoranda on the same, from Dr. D. W. Vance. - 364
3. Remarks on the same by Dr. D. Osgood. - - 365

### MISCELLANEOUS INTELLIGENCE.

### I. ANALECTIC.

1. Ligatures in nævi materni p. 368.—2. Acupuncturation in rheumatism p. 369.—3. Treatment of colica pictonum p. 369.—4. Obstruction in the arteries p. 370.—5. Encephalitis cured by acupuncturation p. 371.—6. Wounds and ulcers cured by plates of lead p. 371.—7. Remarkable diseases of the epiploon p. 373.—8. Laryngitis p. 373.—9. Enlargement of the upper lip p. 375.—10. New mode of dressing fractures p. 376.—11. Physiological and pathological results of extirpation of the kidneys p. 376.—12. Section of the pneumo-gastric nerves p. 377.—13. Effects of cupping glasses on the developement of Vaccine pustules p. 378.

### II. ANALYTICAL.

14. Coffee p. 381.—15. Respiration of cold air in pulmonary diseases p. 383.—16. Blisters to prevent abortion p. 384.—17. Poisoned wounds. Cupping. Pressure. Ligatures p. 386.—17. Prize dissertation on the effects of ardent spirits p. 389.—19. Address on spirituous liquors p. 389.—20. Iodine in several diseases p. 392.

### III. ORIGINAL.

21. Consulting Surgeon to the Cincinnati Eye Infirmary p. 394.—22. Necrology.—Death of Dr. Gall, and of Dr. Pierson p. 394.

### *NO. VIII.*

### ESSAYS AND CASES.

1. Remarks on the Pathological condition of the System, denominated Congestion. By John P. Harrison, M. D. of Louisville, Ky. - - - 395

### REVIEWS.

II. A physiological memoir upon the Brain. By M. Majendie. Read in a public sitting of the Royal Academy of Sciences, on the 16 of June, 1828. From the French, by Joseph Gardener, M. D. - - - 418
III. Account of the Dengue as it appeared in Charleston, S. C. during the Summer of 1828. By S. Henry Dickson, M. D. Professor of the Institutes and Practice of Medicine, in the Medical College of S. C.
On Dengue. By Dr. Isaac Hays.
History of the Dengue in New Orleans. By Dr. Philip J. Dumaresq.
An account of the Dengue Fever, or late epidemic of the West

Indies. By Richard Tuite, M. D. - - 428

To Subscribers. - - - - 442

*NO. IX.*

ESSAYS AND CASES.

I. A History of the Malignant Intermittent Bilious Fever, which prevailed in and about Indianapolis, the capital of the state of Indiana, in the summer and autumn of 1821. By Dr. Samuel Grant Mitchell. - - - 443
II. A fatal case of rupture of the bladder, from Urinary Calculi, in an Ox. By John Cook Bennett. - - 450
Editorial Note containing an account of the analysis of these Calculi. - - - - - - 451
III. An account or the production of Epilepsy from protracted bathing in a pond. By H. George Doyle, M. D. - 454
Remarks by the Editor. - - - - 457

REVIEWS.

IV. Lectures on Anatomy, Surgery, and Pathology; including Observations on the nature and treatment of Local Diseases; Delivered at St. Bartholomew's Hospital. By John Abernethy, F. R. S. - - - - - 459

*NO. X.*

ESSAYS AND CASES.

I. An account of the successful resuscitation of three persons from suspended animation, by submersion in the river Ohio, for twenty or twenty five minutes. By Richard Povall, M. D. - - . 499
II. Observations on Fever and other Diseases of the South and West. By Medicus. - - - 503

REVIEWS.

III. Lectures on Anatomy, Surgery, and Pathology; including observations on the nature and Treatment of Local Diseases. Delivered at St. Bartholomew's Hospital. By John Abernethy, F. R. S. - - - - 514

*NO. XI.*

ESSAYS AND CASES.

I. A summary view of the Ætiology of Bilious Fever. By John P. Harrison, M. D. - - - - 551
II. Facts and Analogies designed to show that Hydrocyanic Acid is probably the cause of Autumnal Fevers. By G. S. B. Hempstead, M. D. - - - - 564

REVIEWS.

III. De la Lithontritie on Broiment de la Pierre dans la Vessie. Par le Docteur Civiale. Avec cinq planches.
Lithontrity, or breaking down the stone in the bladder, &c. 570

MISCELLANEOUS INTELLIGENCE.

ANALECTIC.

1. On the Valves of the Pulmonary veins.—2. Microscopic researches upon the intimate structure of Animal Tissues.—3. Case of disease of the brain, illustrating the functions of the Fifth pair of Nerves.

—4. Case in which there was a diminution of sensibility on one side, without loss of the power of motion; and a loss of muscular power on the other side without any diminution of sensibility.—5. On the effects of the division or organic lesion of the Fifth pair of Nerves.—6. Vision after the destruction of the optic nerves.—7. On the effects of galvanism on the nerves.—pp. 596—603.

ORIGINAL.

First Annual Report of the Visitors of the Cincinnati Eye Infirmary, p. 603. Western Medical Classes, p. 606. Necrology, p. 606.

*XII.*

ESSAYS AND CASES.

Observations on Fever and other Diseases of the South and West. By MEDICUS, No. ii. - - - 607

Cases, illustrating the efficacy of Paracentesis, in Abscesses of the Pleura: - - - - -

1. A case of Empyema successfully treated by Paracentesis. By JEREMIAH H. BROWER, M. D. - - 612

2. An account of a case of Empyema consequent upon Measles, cured by Paracentesis. By the EDITOR. - - 617

3. Notes of a case of Empyema from Chronic Pleurisy, in which the operation gave relief. By the EDITOR. - 620

Notes of a case of imitative Neuralgic affection, occasioned by Mercury. By Dr. J. BENNET. - - 623

A Sketch of the Life and Character of Dr. Thomas Hinde. By the EDITOR. - - - - - 625

MISCELLANEOUS INTELLIGENCE.

I. ANALECTIC.

1. Blisters in the early stages of Measles. p. 635.—2. Mr. Lizars' Method of Amputation, 635.—3. Mercury, 636.—4. Treatment of Dropsies, 636.—5. Puerperal Insanity, 638 —6. Case of Tetanus with Inflammation of the spinal chord, 638.—7. Difference of the Blood in the Veins and Capillary Vessels, 639.—8. Cure of White Swelling, by Frictions of Iodine, 640.—9. Extirpation of the Uterus, 640.—10. On the Coagulation of the Blood, 642.—11. On the Diseases of the Kidneys and Ureters, 642.—12. On the use of Circular Ligatures in Intermittents, 645.—13. On certain methods of treating Chronic Inflammations of the Eye, 645.—14. Remedy for Opacity of the Cornea, 546.—15. Signs of Drowning, 647.

II. ANALYTICAL.

16. Removal of loose substances from the Knee joint, 648.—17. Acetate of Ammonia in Uterine diseases, 649.—18. Hydrocyanite of Iron in Epilepsy and Chorea. 650.—19. Amputation: Circular and Flap Operations, 650.—20. Spina Bifida, 651.—21. Graduates of Transylvania University, 652.—22. Graduates of the Medical College of Ohio, 654.

